# *Fasciola hepatica* coinfection modifies the morphological and immunological features of *Echinococcus granulosus* cysts in cattle

**DOI:** 10.1186/s13567-020-00799-5

**Published:** 2020-06-05

**Authors:** Christian Hidalgo, Caroll Stoore, Marcela Hernández, Rodolfo Paredes

**Affiliations:** 1 Laboratorio de Medicina Veterinaria, Escuela de Medicina Veterinaria, Facultad de Ciencias de la Vida, Universidad Andres Bello, Santiago, Chile; 2 Instituto de Ciencias Agroalimentarias, Animales y Ambientales (ICA3), Universidad de O’Higgins, San Fernando, Chile; 3 Laboratorio de Biología Periodontal y Departamento de Patología y Medicina Oral, Facultad de Odontología, Universidad de Chile, Santiago, Chile

## Abstract

Polyparasitism occurs when animals harbour multiple parasites concomitantly. It is a common occurrence but is generally understudied in wild and domestic animals. *Fasciola hepatica* and *Echinococcus granulosus*, which are helminths of ungulates, frequently coinfect cattle. The effects of this particular type of polyparasitism are not well documented. The metacestode of *Echinococcus granulosus* is surrounded by the adventitial layer, which constitutes the host immune response to the parasite. This layer in cattle is produced by a granulomatous reaction and is involved in echinococcal cyst (EC) fertility. Due to the systemic immune-modulating abilities of *Fasciola hepatica*, coinfection possibly generates a favourable environment for EC growth. A total of 203 *Echinococcus granulosus* sensu stricto cysts were found in 82 cattle, of which 42 ECs were found in 31 animals coinfected with *Fasciola hepatica*. The overall infection intensity was 3 cysts per animal. Coinfection with *Fasciola hepatica* decreased the mean infection intensity to 1.4 cysts per animal. Regarding EC size, coinfection resulted in smaller ECs (15.91 vs 22.09 mm), especially for infertile lung cysts. The adventitial layer of ECs in coinfected animals lacked lymphoid follicles and palisading macrophages, which are generally hallmarks of the granulomatous immune response. The ECs in coinfected animals had organized laminated layers, whereas those in animals without coinfection did not. Although coinfection was not statistically associated with EC fertility, we did not find fertile cysts in the livers of coinfected animals. We concluded that coinfection with *Fasciola hepatica* and *Echinococcus granulosus* has a detrimental effect on ECs, particularly infertile cysts.

## Introduction

Polyparasitism, which is co-infection with different parasite species in the same host, is a well-documented subject in the medical, veterinary, and zoological literature. Most animals in nature and humans in rural areas are concurrent hosts of a wide array of parasites [1]. Although polyparasitism sometimes affects the viability of the parasite within its host or its transmission to other hosts, it is a poorly studied aspect of parasitism, particularly in terms of the synergistic or antagonistic relationship that occurs between two parasites within the same host [2]. These interactions occur when the presence of one parasite creates an environment that facilitates or impedes, direct or indirectly, infection with another parasite [1]. In this way, polyparasitism increases or decreases the susceptibility of the host to other infections, which are sometimes fatal [3–6]. Despite being extremely relevant for the comprehension of the host-parasite relationship, the information found in the literature that illustrates the real effects of polyparasitism on host health is scarce [1]. As many parasitic diseases are also zoonoses, it is an important issue in both animal and human health.

The diverse group of helminth parasites known as “masters of immune modulation” is a group of animals that show various infection strategies that generally induce a “Th2-like” immune response [7–10]. This type of immune response against parasites normally controls the infection but rarely eliminates the parasite. This shows that helminths have developed mechanisms of both evasion and modulation of the immune response by taking advantage of host tolerance and anti-inflammatory mechanisms [8].

Experimental studies of co-infection with parasites with similar characteristics show that they often compete for their ecological niche [11]. Experiments in rats with *Moniliformis moniliformis* and *Hymenolepis diminuta* determined that in single infections, both parasites are localized in the anterior portion of the small intestine, whereas in coinfections, *Hymenolepis diminuta* is pushed to a posterior segment; interestingly, *Hymenolepis diminuta* lives longer than *Moniliformis moniliformis*, and once the latter dies, *Hymenolepis diminuta* moves to its preferred anatomical place [1]. Another example is nematodes from the *Steinernema* and *Heterorhabditis* genera [11].

In cattle, there are two zoonotic parasitic helminths that are frequently found coinfecting the same host: *Echinococcus granulosus* and *Fasciola hepatica*, which are a cestode and a trematode, respectively. *Echinococcus granulosus* has an indirect life cycle [12] in which cattle and other ungulates are intermediate hosts and dogs are definitive hosts. In the intermediate host, the metacestode of *Echinococcus granulosus* develops as cysts in the liver, lungs, and other viscera, causing a disease termed cystic echinococcosis [13]. *Echinococcus* cysts (ECs) are maintained by a syncytium of cells called the germinal layer, which synthesizes an extracellular matrix termed the laminated layer that forms protoscoleces through cellular differentiation processes. Both layers are encapsulated by a third layer of host origin named the adventitial layer [14]. *Fasciola hepatica* also has an indirect life cycle, with cattle as the definitive host. The adult fluke lives in the bile ducts [15].

Two types of *Echinococcus* cysts are found in the intermediate hosts. Fertile ECs, characterized by the presence of protoscoleces, are infective in the definitive host, which enables them to continue their life cycle. Infertile ECs either do not have or have only a few nonviable protoscoleces. The cellular and molecular mechanisms that cause EC infertility remain unknown [16]. We previously reported that cattle age is not associated with EC fertility [17]. Studies point towards the local immune response as the most likely cause [18–23]. In cattle, the EC adventitial layer is characterized by a granulomatous reaction with an accumulation of monocytic cells, which are believed to be in control of isolating and destroying a persistent foreign body. The hallmarks of this reaction are two special kinds of macrophages called epithelioid cells and giant multinucleated cells [24]. Studies on cattle that describe their cellular components refer to the adventitial layer as the host-derived fibrous capsule [25–32]. One study of fertile liver cysts found a significant number of B cells and some polymorphonuclear cells and monocytes [25]. Another study compared ECs between sheep and macropods, reporting that fertile cysts have either foamy macrophages and giant multinucleated cells or fibrous granulation tissue devoid of cells [27]. The authors pondered whether fertile cysts develop under inflammatory conditions. In the case of infertile cysts, Sakamoto and Cabrera [29] described the cellular components of the adventitial layer of infertile cysts with lymphocytes, macrophages, granulocytes, and giant multinucleated cells. They also reported that cyst size is correlated with the type of cells present in the adventitial layer: small infertile cysts are surrounded by macrophages, while larger cysts are surrounded by eosinophils.

Although *Echinococcus granulosus* and *Fasciola hepatica* belong to different taxonomical categories, they have common antigens. Sera from animals experimentally infected with *Fasciola hepatica* or *Echinococcus granulosus* recognize antigens from *Schistosoma mansoni*. When rabbits are immunized with *Echinococcus granulosus* antigens, their sera recognize the 205, 166, 45 and 32 kDa proteins of *Fasciola gigantica*, as shown by a Western blot assay. Likewise, when animals are immunized with *Fasciola gigantica* antigens, their sera recognize *Echinococcus granulosus* the 205, 148 and 32 kDa protoscolex antigens [33].

Th2-like immune modulation induced by both *Fasciola hepatica* and *Echinococcus granulosus*, along with their similar immune modulation strategies, should favour the development of both parasites. However, cattle with both *Fasciola hepatica* and *Echinococcus granulosus* have an decreased proportion of liver cysts and an increased proportion of small lung cysts [17]. It is possible that this non-specific occurrence is due to liver damage caused by *Fasciola hepatica*. However, the increased proportion of small lung cysts suggests a systemic effect exerted by *Fasciola hepatica* infection. These systemic effects in coinfections with other pathogens are well studied. *Fasciola hepatica* inhibits the T_H_1 response against *Bordetella pertussis* [34] and inhibits the cellular immune response against *Mycobacterium tuberculosis* [35]. This research article suggests that *Fasciola hepatica* coinfection has a systemic effect on the *Echinococcus granulosus* metacestode, which affects both the number and size of ECs present in coinfected animals. Our study provides a deeper characterization of the adventitial layer of the EC and the way in which *Fasciola hepatica* coinfection affects the local immune response against *Echinococcus granulosus*.

## Materials and methods

### Sample collection

Samples were collected from abattoirs at three different geographical locations in the Metropolitan (33.4489° S, 70.6693° W), Los Lagos (40.5762° S, 73.1149° W) and Magallanes (53.1638° S, 70.9171° W) regions over a seven-day period at each location. According to local animal health authorities, more than 90% of viscera confiscation in cattle is caused by either *Fasciola hepatica* and/or *Echinococcus granulosus* [36]. During the slaughtering process, all livers were inspected for *Fasciola hepatica* and *Echinococcus granulosus* infections, while lungs were inspected only for *Echinococcus granulosus* infection. *Fasciola hepatica* infections were determined as previously described [17]. Only adult animals (> 2 years) were sampled. Each EC was obtained from the viscera, placed into a labelled sealed bag and stored in an isothermal container. Each bag represented all the ECs obtained from each slaughtered animal with or without coinfection. All sampling procedures were approved by the Universidad Andres Bello Bioethics Committee (protocol number 016/2016).

### Sample processing

**Figure 1:**
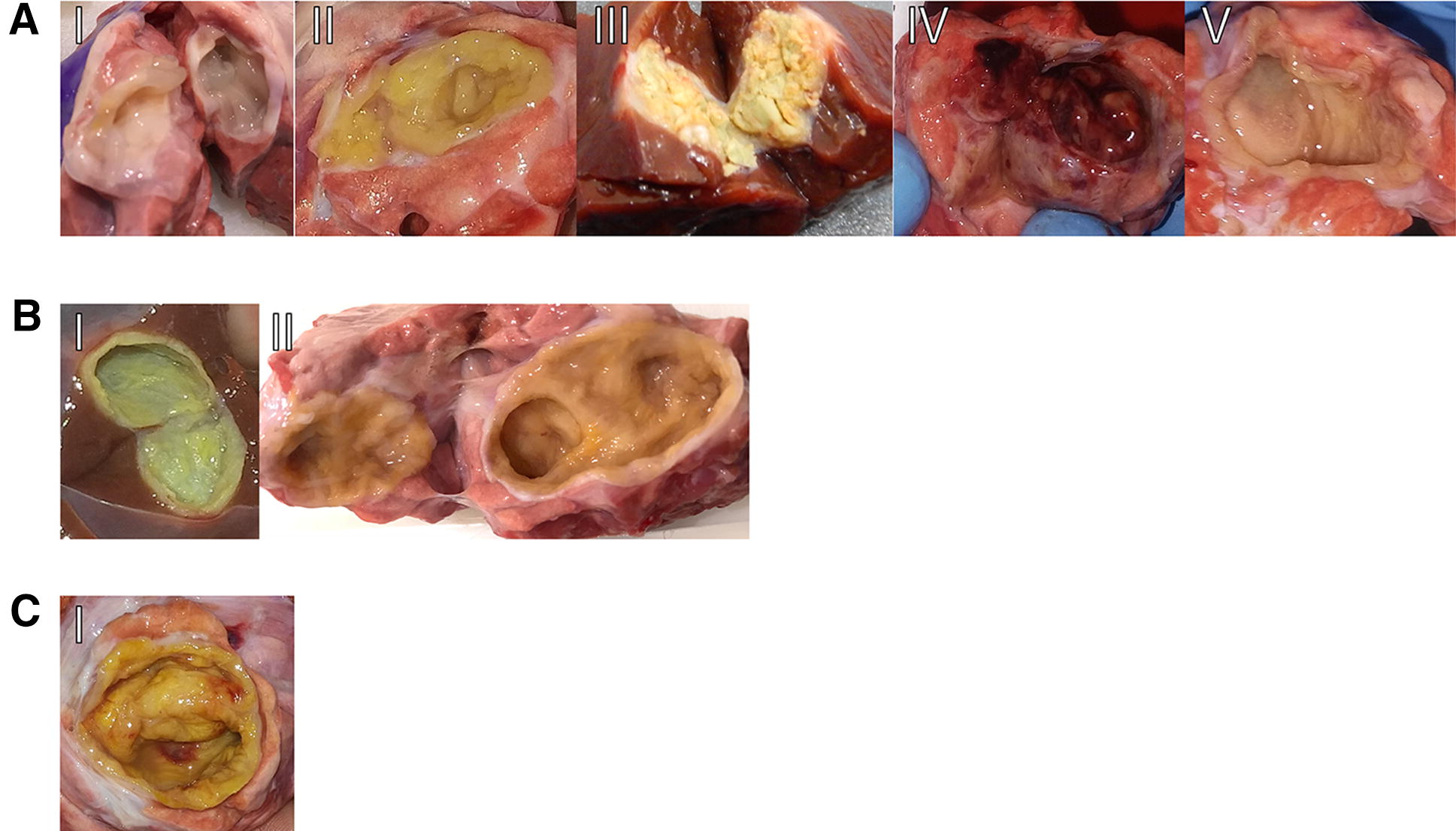
**Gross echinococcal cyst features**. **A** Representative images of white (I), yellow (II), calcified (III), haemorrhagic (IV) and mixed white/yellow (V) germinal/laminated layer colours. **B** Representative images of ECs with one chamber (I) or multiple chambers (II). **C** Representative image of a yellow-coloured EC with a haemorrhagic patch. EC: *Echinococcus* cyst.

In the laboratory, each cyst was individually identified with a corresponding number identifying the organ (liver or lung) and the host (single or multiple cysts) and measured with a digital caliper. Using a sterile syringe, the hydatid fluid was removed and stored. Using a disposable scalpel, the cyst was opened along the longest longitudinal axis, and fertility was assessed as previously described [37]. EC fertility was determined by the presence of a white laminated layer that easily detaches from the adventitial layer and protoscoleces floating in the remaining hydatid fluid and/or attached to the inside of the cyst. ECs with a yellow-ochre laminated layer firmly attached to the adventitial layer with clear hydatid fluid were considered infertile. Suspicious ECs were examined under a light microscope for fertility assessment (i.e., those with a whitish laminated layer without protoscoleces floating in the remaining fluid or attached to the inside of the cyst). Gross EC features were also recorded. Five different germinal/laminated layer colours were recognized: white, yellow, calcified, haemorrhagic, and mixed white/yellow. ECs presented as either one chamber or multiple chambers. Regardless of colour, some ECs presented patches of haemorrhage. These classifications are depicted in Figure 1.

### DNA processing and analysis

To determine the *Echinococcus granulosus* species, the full length of the mitochondrial cox1 gene was amplified and sequenced as previously described [38, 39]. Briefly, DNA was extracted using a commercial kit (Promega ReliaPrep gDNA Tissue Miniprep). Fifty nanograms of DNA was used for PCR, and products were visualized in 1% agarose gels with SYBRsafe stain. Sample sequencing was performed with 50 μL of PCR product and two primers. Finally, both the forward and reverse sequencing data were imported into Geneious software and aligned using the Eg01 haplotype sequence as a template (Accession No. JQ250806). Only samples that represented *Echinococcus granulosus* sensu stricto were included in this study.

### Histological processing and analysis

Samples were processed as previously described [40]. Briefly, fixed samples were cut, oriented transversally and inserted into biopsy cassettes. Paraffin embedding was performed in a Leica Tissue Processing station using a 12-h protocol. Paraffin blocks were made using a Leica inclusion station. Afterwards, each block was cut into 5-μm tissue sections using a Leica microtome. Each slide was stained with haematoxylin and eosin (HE). For each cyst sample, two slides were examined under an inverted light microscope (Olympus FSX100), and both the laminated and adventitial layers were analysed.

An image of each slide was obtained at low-power magnification that contained a total length of 4 to 5 mm of the laminated layer. Ten measurements of thickness were made across the whole length of the sample.

### Adventitial layer morphohistological analysis

**Figure 2:**
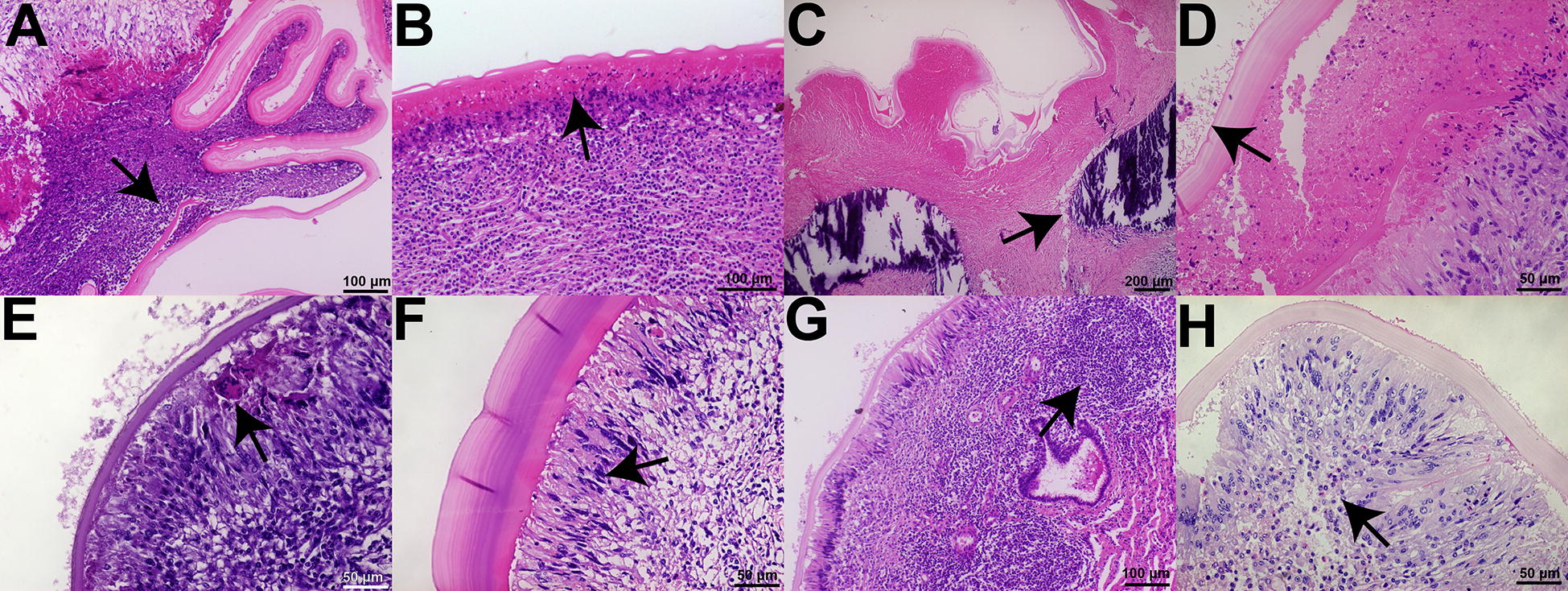
**Adventitial layer histological features**. Histological sections of the adventitial layer of cattle ECs with different features. **A** Laminated layer disorganization. **B** Necrosis between the laminated and adventitial layers. **C** Calcifications. **D** Host cells in the germinal layer. **E** Giant multinucleated cells. **F** Palisading macrophages. **G** Lymphoid follicles. **H** Eosinophils. Arrows mark indicated feature. Stain: haematoxylin and eosin. EC: *Echinococcus* cyst.

The criteria used to determine if a histological feature was present in each slide are found in Table 1. A representative image of each feature is shown in Figure  2.

**Table 1:** **Criteria for determining the presence of the histological characteristics of the adventitial layer.**

	Histological feature	Criteria
Laminated layer disorganization	Either whole laminated segments inside the adventitial layer or host cell infiltration between the laminated layer sheets	At least one in the entire sample
Necrosis between the laminated and adventitial layer	Presence of an eosinophilic zone devoid of cells without a fibrous structure	Across one low-power magnification field (approximately 5 mm)
Calcifications	Presence of strong haematoxylin staining without cells and with crystal structures	At least one in the entire sample
Host cells in the germinal layer	As previously described [42]	At least one in the entire sample
Giant multinucleated cells	Cells with 5 or more nuclei near the laminated layer	At least one per high-power magnification field (1 mm)
Palisading macrophages	The presence of foamy macrophages with cytoplasmic projections in contact with the laminated layer	Across one low-power magnification field
Lymphoid follicles	Circular structures with lymphocytes near the laminated layer	At least one per low-power magnification field
Eosinophils	Cells with eosinophilic cytoplasm near the laminated layer	At least ten or more eosinophil nuclei per high-power magnification field

When a feature was not recorded in both slides, it was considered absent.

### Statistical analysis

All data were recorded and analysed in both GraphPad Prism version 8.00 for Mac OSX, (GraphPad Software, La Jolla, USA) and Stata v12. Data distribution was assessed by the Shapiro–Wilk test. Quantitative nonnormally distributed data and ordinal variables are presented as the median and IQR, and nonparametric Mann–Whitney tests were performed. The Chi square test was used for determining the statistical associations between nominal variables. *P* values below the 0.05 threshold were considered statistically significant.

## Results

### Global cattle coinfection status

**Figure 3:**
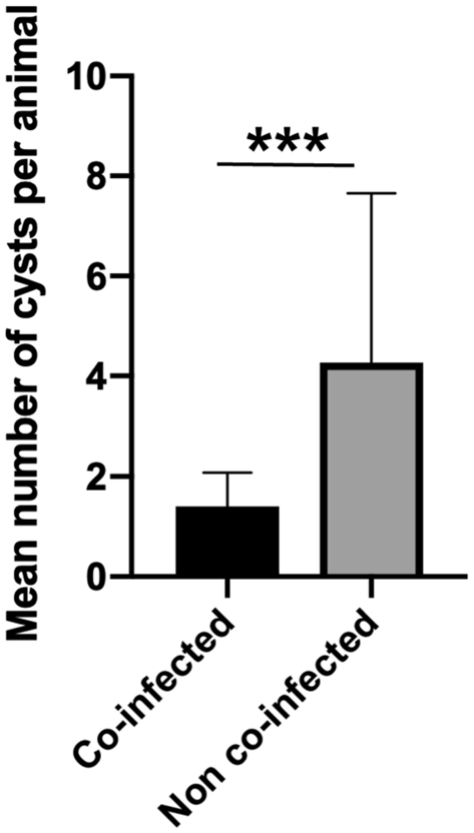
**Intensity of*Echinococcus granulosus*infection in cattle coinfected with*Fasciola hepatica***. The results are expressed as the mean number of ECs per animal ± standard deviation. Statistical significance at *p* < 0.05, **p < 0.01 ***p < 0.001. EC: *Echinococcus* cyst.

A total of 204 ECs were found in 83 cattle. After molecular identification, only one EC was determined to be *E. ortleppi* and was removed from the study, bringing the total number of samples to 203. Of these, 42 ECs (20.6%) were found in 31 animals coinfected with *Fasciola hepatica* (15 liver cysts and 27 lung cysts). The overall infection intensity was 3 cysts per animal. Cattle coinfected with *Fasciola hepatica* had a mean infection intensity of 1.4 cysts per animal. Non-co-infected animals had a significantly higher mean infection intensity of 4.2 cysts per animal (*p* = 0.0006) (Figure  3).

### Co-infection and EC fertility

No fertile liver ECs were found in animals coinfected with *Fasciola hepatica*. There was no statistical association between the presence of *Fasciola hepatica* and EC fertility in the lungs (Fisher’s exact test, *p* = 0.35) or liver (Fisher’s exact test, *p* > 0.99). The numbers of fertile and infertile cysts are summarized in Table 2.

**Table 2:** **Fertility of ECs from both the liver and lungs of cattle coinfected with*Fasciola hepatica.***

	Liver		Lungs		Total	
	Fertile	Infertile	Fertile	Infertile	Fertile	Infertile
Co-infected	0	15	3	24	3	39
Non-co-infected	1	70	4	86	5	156

EC: *Echinococcus* cyst.

### Coinfection and EC size

**Figure 4:**
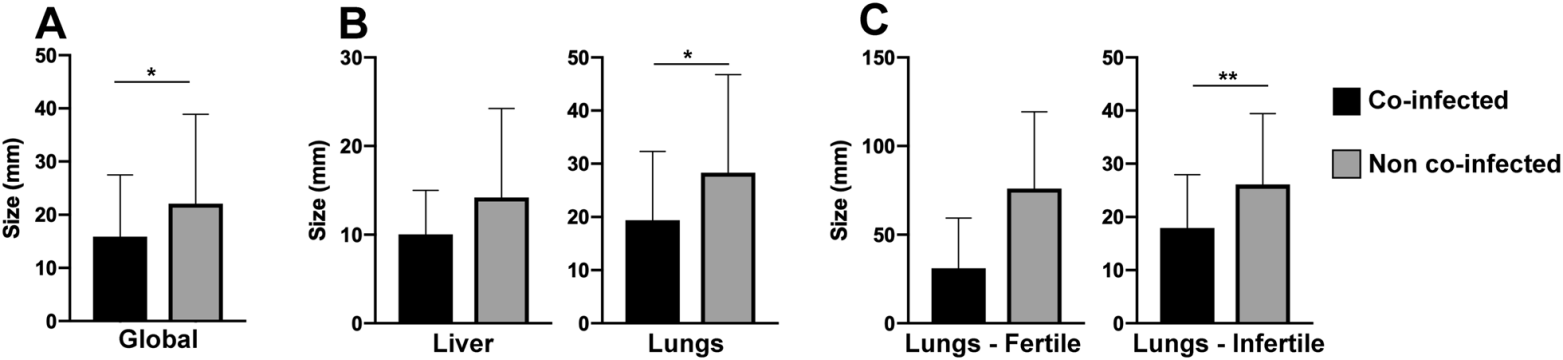
**EC mean diameter in cattle with or without*Fasciola hepatica*coinfection**. **A** Global difference in size; coinfected animals had smaller EC*s*. **B** Difference between liver and lung ECs; coinfection was associated with smaller lung cysts. **C** Difference between fertile and infertile lung ECs; infertile cysts were smaller in coinfected animals. The results are expressed as the mean size of ECs ± standard deviation. Statistical significance at *p* < 0.05, **p < 0.01 ***p < 0.001. EC: *Echinococcus* cyst.

The mean diameter of ECs in animals with *Fasciola hepatica* coinfection (15.91 mm) was significantly smaller (*p* = 0.0243) than that of ECs in animals without coinfection (22.09 mm) (Figure  4A). When analysed according to organ location, the ECs in lungs in co-infected animals were smaller (19.38 mm) than ECs in non-co-infected animals (28.29 mm) (*p* = 0.0212) (Figure  4B). When analysed according to fertility, infertile ECs were smaller (17.92 mm) in coinfected animals than those in non-co-infected (26.07 mm) animals (*p* = 0.0066) (Figure  4C).

### Coinfection and gross hydatid cyst features

There were no statistical associations between *Fasciola hepatica* coinfection and the presence of haemorrhagic patches or the presence of single or multiple chambers in ECs. Regarding the inner chamber colour, *Fasciola hepatica* coinfection was associated with an overall decreased proportion of cysts with the mixed yellow/white phenotype (*p* = 0.0082), and only one case of a cyst with a haemorrhagic inner chamber was recorded. These results are summarized in Table 3.

**Table 3:** **Distribution of the inner chamber colours of*Echinococcus*cysts according to*Fasciola hepatica*coinfection.**

	Inner chamber colour					
	White	Yellow	Calcified	Haemorrhagic	Mixed	Total
Co-Infected	6	18	4	1	5	34
Non-Co-Infected	6	101	12	0	28	147
Total	12	119	16	1	33	181

### Co-infection and adventitial layer features

The adventitial layer of 185 ECs was analyzed (twelve liver and 23 lung cysts from coinfected animals and 62 liver and 88 lung cysts from non-coinfected animals). In each sample, *Fasciola hepatica* coinfection was associated with the presence or absence of the following inflammatory features: laminated layer disorganization, necrosis between the laminated layer and adventitial layer, calcifications, host cells infiltrating the germinal layer, giant multinucleated cells, palisading macrophages, lymphoid follicles, and eosinophils.

Coinfection was associated with an absence of laminated layer disorganization (*p* = 0.0043), palisading macrophages (*p* = 0.0009), and lymphoid follicles in the adventitial layer (*p* = 0.0003). The results are summarized in Table 4. Regarding *Fasciola hepatica* coinfection and the laminated layer thickness, there was no association of fertile or infertile cysts with thinner or thicker laminated layers.

**Table 4:** **Presence or absence of adventitial layer features of ECs with and without*Fasciola hepatica*coinfection.**

	Laminated layer disorganization		Necrosis between the laminated and adventitial layer	
	Absence	Presence	Absence	Presence
Co-infected	24**	11**	13	22
Non-co-infected	61**	89**	39	111

	Calcifications		Host cells in the germinal layer	
	Absence	Presence	Absence	Presence
Co-infected	30	5	26	9
Non-co-infected	108	42	102	48

	Giant multinucleated cells		Palisading macrophages	
	Absence	Presence	Absence	Presence
Co-infected	2	33	7***	28***
Non-co-infected	13	137	4***	146***

	Lymphoid follicles		Eosinophils	
	Absence	Presence	Absence	Presence
Co-infected	10***	25***	2	33
Non-co-infected	8***	142***	1	149

EC: *Echinococcus* cyst.

Statistical significance at *p* < 0.05, **p < 0.01 ***p < 0.001, Fisher’s exact test.

In summary, *Fasciola hepatica* coinfection had a paradoxical effect on ECs, as it was associated with the absence of fertile ECs in the liver, decreased infection intensity, and decreased EC size. However, it was also associated with an absence of inflammatory features in the adventitial layer, such as laminated layer disorganization, palisading macrophages, and lymphoid follicles.

## Discussion

Polyparasitism of *Fasciola hepatica* and *Echinococcus granulosus* is a common occurrence that is not often reported in scientific studies. A previous study found that 46.57% of *E. granulosus*-infected cattle harbour both parasites simultaneously [17], which was a higher percentage than that found this report. We found that *F. hepatica* infection also decreases the *E. granulosus* infection intensity. This leads to the overrepresentation of the percentage of ECs in non-co-infected animals, as these animals harbour a higher number of ECs than co-infected animals. Additionally, fertile liver ECs were only found in the absence of *Fasciola hepatica* infection. While this observation suggests a detrimental effect of *Fasciola hepatica* on the *E. granulosus* metacestode, coinfected and non-coinfected cattle did not show significant differences in the fertility of ECs in the liver and lungs. The cause of this effect is not known. Due to liver damage caused by both acute and chronic *Fasciola hepatica* infection [41], it is plausible that *Echinococcus granulosus* oncospheres are unable to thrive in the liver, and only those that travel via lymph towards the lungs are able to fully develop [17]. This may explain the decreased intensity of infection observed in naturally coinfected cattle. Another variable that affects the intensity of infection is weather. It also directly affects oncosphere viability. Higher temperatures and direct sunlight confer oncospheres the ability to infect intermediate hosts [42]. One of the regions that was sampled may confound our results, as it has both optimal climatic conditions for oncosphere survival and is free of *Fasciola hepatica*. Exclusion of the samples from this region from the analysis did not affect our results. Notably, regardless of coinfection status, the overall intensity of infection in this study (3 cysts per animal) is lower than that reported for cattle elsewhere [43, 44].

Cattle EC size varies across studies and the type of viscera. One study showed that lung cysts are larger than liver cysts (mean diameters of 43.1 mm and 35.2 mm, respectively) [44]. A second study showed cyst sizes ranging from 4 to 108 mm, with most cysts measuring between 11 and 50 mm [45]. Both studies reported a higher intensity of infection in the lungs than in the liver but did not mention coinfections with other parasitic diseases. Our results show that lung ECs are larger than liver cysts. *Fasciola hepatica* coinfection is associated with smaller cyst sizes, specifically for infertile lung cysts. On average, infertile lung cysts were half the size in coinfected animals compared to those in non-coinfected animals. To the best of our knowledge, this is the first study that associates cattle hydatid cyst size with *Fasciola hepatica* coinfection.

EC fertility is associated with the intensity of the inflammatory reaction in the adventitial layer [40], and *Fasciola hepatica* modifies the cattle systemic immune response [35]. We hypothesized that adventitial layer characteristics are associated with coinfection status. This study shows that coinfection is associated with an absence of both lymphoid follicles and laminated layer disorganization in the adventitial layer of liver ECs. Lung ECs frequently show an absence of calcification, necrosis under the laminated layer, and palisading macrophages of the adventitial layer. These findings suggest a positive environment for EC growth, as the presence of these histological features is the hallmark of the granulomatous reaction associated with EC infertility [40]. *Fasciola hepatica* modulates host macrophages, stimulates fibrosis, and inhibits TLR3 and TLR4 activity (both of which are T_H_1-associated receptors and thus are detrimental for EC survival) [46]. This is a possible mechanism by which the granulomatous reaction is diminished. Overall, *Fasciola hepatica* coinfection is associated with weaker inflammatory responses in the adventitial layer. Nonetheless, it is also associated with decreased numbers of cysts and intensities of infection and an absence of fertile liver ECs.

We could not prove whether polyparasitism between *Fasciola hepatica* and *Echinococcus granulosus* was synergistic or antagonistic. Echinococcal cyst samples were obtained at abattoirs from naturally infected animals. It is not possible to determine which parasite enters the host first. *Fasciola hepatica* adult worms were not evaluated. We only sampled cattle with cystic echinococcosis only and coinfection but did not include cattle infected only with *Fasciola hepatica*. Further experimental studies are needed to better define the interaction involved in coinfections.

## Data Availability

All data generated or analysed during this study are included in this published article [and its supplementary information files].
